# Acetabular Fracture in a Skeletally Mature Patient With Osteogenesis Imperfecta Treated With Open Reduction Internal Fixation: A Case Report

**DOI:** 10.7759/cureus.50394

**Published:** 2023-12-12

**Authors:** Mohamed A Alsehly, Mohammed A Althagafi, Siyad A Alfaraidy

**Affiliations:** 1 Department of Orthopaedic Surgery, King Faisal Specialist Hospital and Research Centre, Riyadh, SAU; 2 Department of Orthopaedic Surgery, Alfaisal University College of Medicine, Riyadh, SAU; 3 Department of Orthopaedic Surgery, King Saud Medical City, Riyadh, SAU

**Keywords:** judet–letournel, bone fragility, acetabular fracture, open reduction internal fixation, osteogenesis imperfecta

## Abstract

Patients with osteogenesis imperfecta often present with and are managed for various fractures given the brittle bones associated with this disease. Acetabular fractures are one of the most complicated presentations and management is often strenuous on both the patient and the treating surgeon. There is a lack of evidence on how to approach these patients and not many cases reported in the literature. Open reduction and internal fixation can be successful for these patients given extra care is undergone to protect the patient’s increased risk of intra-operative and post-operative complications, and a thorough understanding of the pathophysiology of this disease.

## Introduction

Osteogenesis imperfecta (OI) is a heritable systemic disorder of bone and connective tissue characterized by bone fragility leading to skeletal deformities in more severe cases, blue sclerae, hearing loss, and short stature, due to the structural weakness of bones. Mutations in the COL1A1 and COL1A2 genes lead to deficient synthesis or processing of type 1 collagen, an important component that contributes to providing bones with their mechanical properties of strength and flexibility, leading to defective bone matrix formation [1]. This deficiency manifests as what is called "brittle bones", bones that break easily by low energy trauma. Collagen Type 1 also gives blood vessels strength, therefore, OI patients’ tend to have higher tendencies for easy bruising and intraoperative bleeding. Studies revealed increased capillary fragility, prolonged bleeding time and abnormal prothrombin consumption. [2]. Therefore, patient with OI that sustain acetabulum fractures present a dilemma for the treating physician, whether to take the patient for operative fixation and deal with the possible complications mentioned or treat the patient in a conservative manner. We present a case of a skeletally mature patient with OI who sustained an acetabulum fracture and was successfully treated with open reduction internal fixation.

## Case presentation

A 16-year-old female, known to have OI Sillence type 1 [3], presented with left hip pain after a fall on her left side. The patient followed in our hospital since the age of three years for management of her brittle bone with bisphosphonates and for her short stature with the Endocrinology team. As well as having undergone posterior instrumented spinal fusion for her scoliosis and multiple corrective osteotomies and Fassier-Duval nail insertions in both femurs.

**Figure 1:**
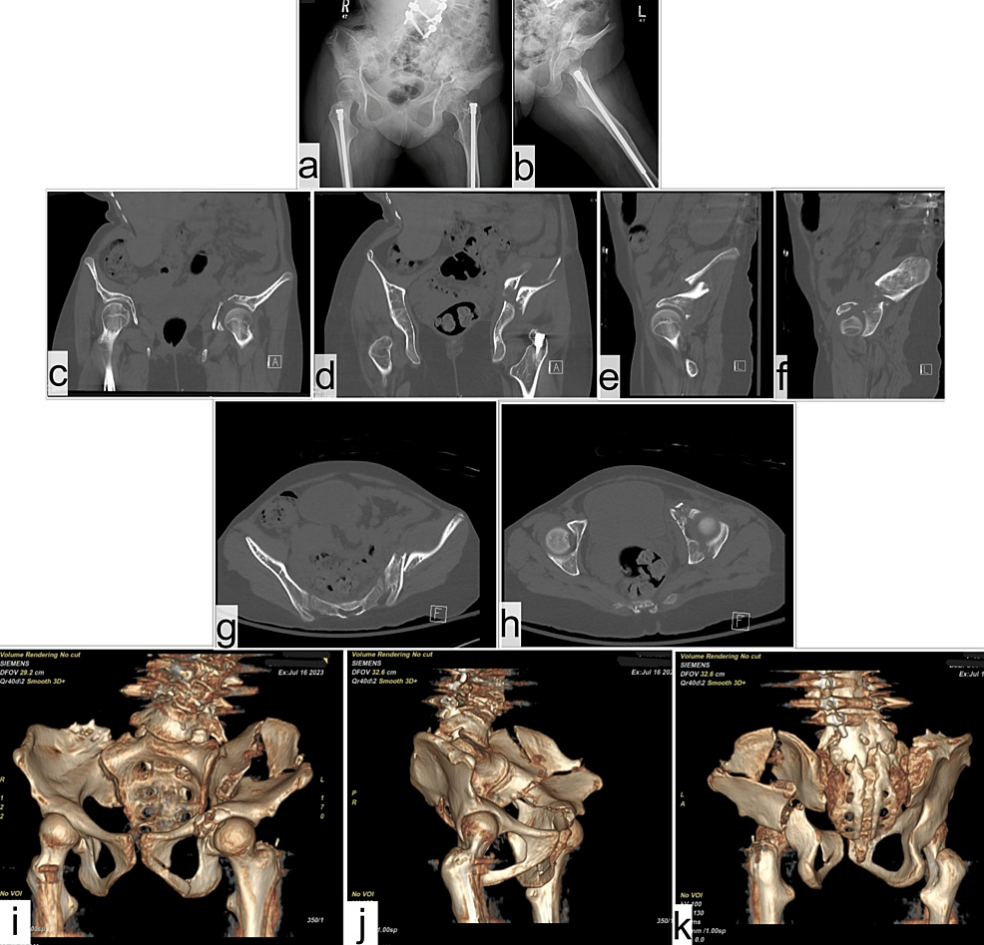
Preoperative imaging showing X-rays (A: AP pelvis, B: lateral left hip), and CT scan (C,D: coronal) (E,F: sagittal), (G,H: axial), (I,J,K: 3D reconstruction) showing both column associated fracture of left acetabulum with involvement of the iliac wing. AP: anterior-posterior

She initially presented in another hospital at the age of 16 in her local city with a history of trauma to her left side after falling from steps and was referred to our facility for definitive management. Upon presentation the patient was vitally stable, had left hip pain, as well as tenderness, mild swelling, and restricted range of motion due to pain around the left hip, and inability to bear weight. Initial imaging studies (Figure 1) showed a comminuted fracture involving the left hemipelvis with involvement of the anterior and posterior wall of the acetabulum as well as the left iliac wing and associated with a soft tissue hematoma adjacent to the fracture site. No other associated injuries were documented. The fracture was diagnosed as associated both column fracture according to the Judet-Letournel classification system.

Informed consent was taken from the patient’s father after a thorough discussion, and she was prepared for surgery. The patient was taken to surgery 17 days after the injury occurred.

Patient was put in supine position, under general anesthesia, after prepping and draping of the pelvic area, we used modified Stoppa approach midline Pfannenstiel incision two fingerbreadths proximal to the pubic bone with an about 8 cm length skin incision. Dissection of subcutaneous tissue with deep fascia was done. Linea alba was opened in line with a skin incision. Bladder was protected and retracted. Then, retroperitoneal dissection over the pelvic brim was done. We then packed the area and then opened a lateral window.

Over the iliac crest, we opened about 10 cm skin incision and dissected subcutaneous tissue. Deep fascia was opened in line with the skin incision. Subperiosteal dissection and elevation of the insertion of the abdominal muscle to the iliac crest was done. Then, we packed the lateral window and we were back working from the medial window. Subperiosteal dissection over the pubic bone was done. Fracture site was identified over the pubic bone. All the structure of intrapelvic or abdominal structure was pushed distally in the pelvic area. We found bowel coming in the field. We retracted the bowel, proceeded with our procedure, and contacted the General Surgery team. Fracture site debridement from the medial window was done. Then, we went from the lateral window. Also debridement of the area was done, and anatomical reduction of the iliac bone was done, and stabilized with a reconstruction plate with two screws proximally and two screws distally. Then, we tried to fix the pubic bone and the iliac bone. The patient developed comminution and fracture in the pubic bone. The bone was very brittle.

**Figure 2:**
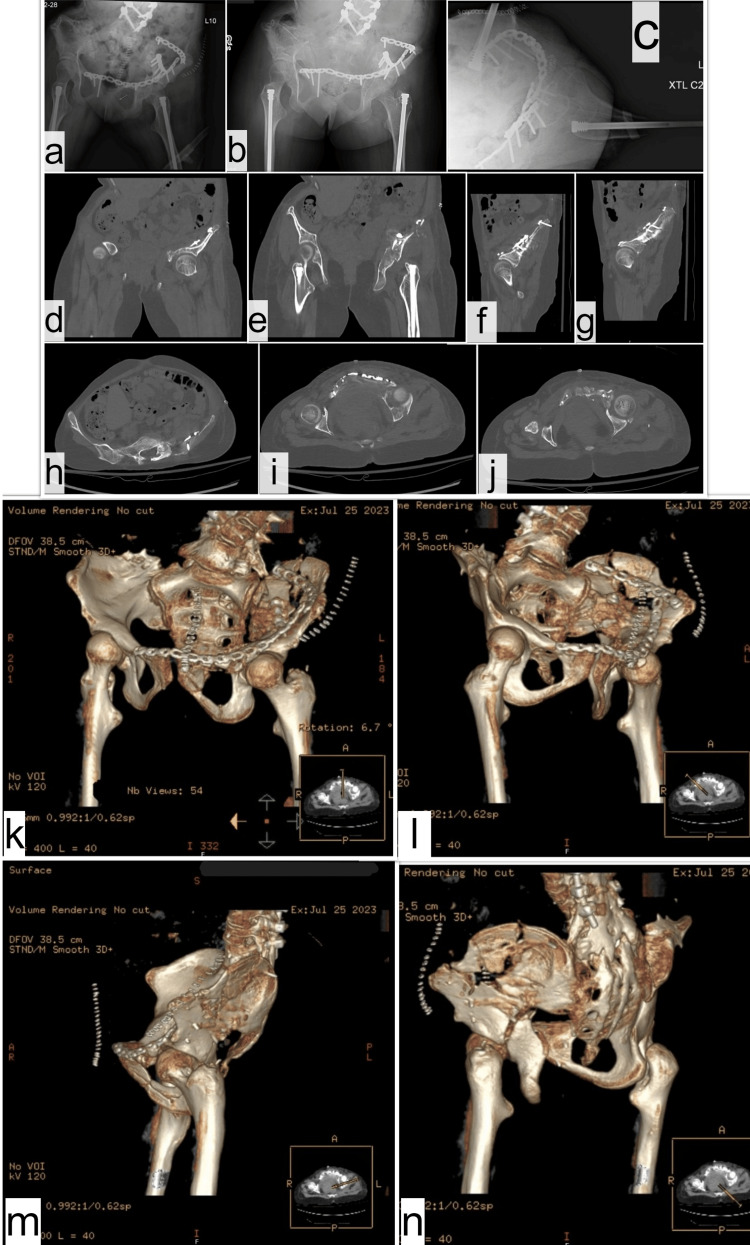
Postoperative imaging showing X-rays (A,B: AP pelvis), (C: lateral left hip), and CT scan (D,E: coronal), (F,G: sagittal), (H,I,J: axial), (K,L,M,N: 3D reconstruction) of reduced left-sided acetabulum fracture. AP: anterior-posterior

We extended our dissection over the right pubic bone and we used a 14-hole reconstruction plate to stabilize the pubic bone to the ileum crossing the symphysis pubis. We used a locking plate. Three screws were applied over the pubic bone. Two screws were applied in the left pubic bone, then two screws were applied in the ileum. There was a comminution in the ileum and the bone was very brittle. We used a locking plate to stabilize the proximal iliac bone fracture through the distal iliac bone fracture, we used a locking plate and one screw was applied as a locking screw proximally and two screws distally. Then, General Surgery identified the perforation in the peritoneum. All bowels were intact. After reduction of the bowel, they closed the peritoneum and they reinforced the area with a mesh, as the soft tissue of the patient was very fragile, after insertion of the mesh, it was stabilized with the glue. Then, irrigation was done. The patient had lost around 3 liters of blood intra-operatively. Two drains were inserted over the lateral wound and medial wound. The wounds were closed in layers (Figure 2).

Postoperative protocol and follow-up

We started the patient with hemoglobin of 9 and transfused four units packed red blood cells intra-op. The patient was kept in the hospital postoperatively for observation and rehabilitation. Her postoperative hemoglobin was 12. She was kept on strict bed rest for two weeks then bed to chair mobilization on a recliner wheelchair for another month then non-weight-bearing mobilization to the left side was started. Three weeks post-op the surgical clips were removed with a healed surgical scar.

She came to our clinic two months after the surgery doing well; her surgical wound has healed, with no swelling, erythema, or tenderness. She was progressed to full weight-bearing regimen three months postoperatively under supervision of a physical therapist in our hospital, and is currently mobilizing fully, comparable to her preoperative state.

## Discussion

For acetabular fractures in OI patients the decision to go for operative fixation is a daunting one. The approach must be individualized based on several factors, including but not limited to the type of fracture, associated injured structures, ambulatory status before the injury, and articular surface involvement which will influence the functional outcome postoperatively [4]. The decision becomes more difficult given the weak status of the patient’s bone and their higher risk of bleeding [5] and implant complications. Pelvis fractures involving the acetabulum in a previously ambulatory, young patient, with normal life expectancy [3], make conservative management a rare option unless the patient is unfit for surgery.

The use of bone graft and bone morphogenetic proteins in patients with OI and pelvic fractures has been reported with good outcomes to add structural support and its osteoinductive properties that enhance bone healing [6] in a previous case report, but we did not use either. Medici et al. [7] concluded that the goal is to restore near anatomic reduction of the acetabulum and give the patient a chance to return to pre-injury mobilization status and to delay the need for hip reconstruction given the patient’s young age. This was our primary goal.

Many difficulties were faced during the fixation with this patient given her brittle bone. Too forceful a reduction or traction risked further fracture propagation, so gentle reduction to near anatomical in the quadrilateral plate and acetabulum was done. Screw purchase was acceptable, but overtightening was a risk for loosening and loss of threads. In previous OI acetabulum fracture cases, protrusio acetabulae or a very thin quadrilateral plate is encountered, which may not permit screw fixation [8]. This is the reason to opt for indirect reduction if possible and plating. In a previous report by Pesch et al. [9] an intramedullary augmentation technique (photodynamic bone stabilization (PBS)) was used to augment established implants in surgical fracture treatment of OI patients. Liprace et al. showed that advanced age and impaction on both the acetabular and femoral head, primary total hip arthroplasty (THA) with bone fixation to support overall construct our patient was suitable for an acute setting [10]. Another complication that may be faced and was reported by Ziran et al. [11] is femoral artery thrombosis and in OI patients with acetabular or pelvic fractures, they recommended avoiding any retraction on the neurovascular bundle, which was stressed during our procedure.

There are limitations to this study; first a longer follow-up is necessary to assess long-term functional outcomes and arthritic changes, as well as more cases to fully understand the difficulties in managing such patients.

## Conclusions

Management of acetabular fracture in OI patients is a daunting task. Many considerations must be taken into account including the age of the patient, the baseline mobilization status, and ability to reconstruct the joint anatomically. Many adjuncts to assist in the surgical fixation of these types of patients have been proposed in the literature. However, further research is needed to better understand the pathophysiology in these types of patients and develop a guideline to improve future outcomes.
